# Determination of mycotoxins, alkaloids, phytochemicals, antioxidants and cytotoxicity in Asiatic ginseng (Ashwagandha, Dong quai, *Panax ginseng*)

**DOI:** 10.1007/s11696-016-0028-0

**Published:** 2016-12-09

**Authors:** Anna Filipiak-Szok, M. Kurzawa, E. Szłyk, M. Twarużek, A. Błajet-Kosicka, J. Grajewski

**Affiliations:** 1 Faculty of Chemistry, Nicolaus Copernicus University in Toruń, Gagarin 7 St., 87-100 Toruń, Poland; 2 Department of Physiology and Toxicology, Faculty of Natural Sciences, Institute of Experimental Biology, Kazimierz Wielki University, Chodkiewicza 30 Str., Bydgoszcz, Poland

**Keywords:** Mycotoxins, Alkaloids, Phytochemicals, Antioxidants, Cytotoxicity, Ginseng, Dietary supplements

## Abstract

Mycotoxins and selected hazardous alkaloids in the medicinal plants (Panax *ginseng*, *Angelica sinensis*, *and Withania somnifera*) and dietary supplements were determined. Purine alkaloids were found in majority of samples; however, isoquinoline alkaloids were less abundant than indole. The predominant alkaloids appear to be caffeine (purine group), harman (indole group) and berberine (isoquinoline). Examined medicinal plants and dietary supplements were contaminated by mycotoxins (especially ochratoxin A 1.72–5.83 µg kg^−1^), and many species of mold (e.g. *Cladosporium*, *Eurotium*, *Aspergillus*, *Rhizopus*, *Penicillium*). MTT cytotoxicity tests revealed that plant and supplements extracts exhibited medium or high cytotoxicity (only Dong quai—low). Moreover, antioxidant activity, total phenolics content and selected phytochemicals were analyzed by spectrophotometric and chromatographic methods. Quercetin and rutin were predominant flavonols (1.94-9.51 and 2.20–7.28 mg 100 g^−1^, respectively). Analysis of phenolic acids revealed—gallic acid, as the most abundant, except *Panax ginseng*, where ferulic acid was prevailing. The results were analyzed by chemometric methods (cluster analysis, ANOVA).

## Introduction

Among studied plant samples, *Withania somnifera* (Ashwagandha, Indian ginseng, poison gooseberry or winter cherry) is cultivated in India, Nepal, China and Yemen (Mirjalili et al. 2009), whereas *Angelica sinensis* (Dong quai, Chinese angelica, Danggui, Tan kue bai zhi, Tang kuei) is a herb indigenous to China (Ross 2001), and Panax ginseng C.A. Meyer (Korean ginseng) is cultivated in Korea and northeast China. The latter one is an herbal root and has been used extensively as Chinese medicine and/or a functional food for centuries (Jung et al. 2002).

Ashwagandha is commonly used as a domestic remedy for several diseases in India as well as other parts of the world (Patwardhan et al. 1998, Mirjalili et al. 2009). Plant is sources of antioxidants (vitamin E, vitamin C, polyphenols including phenolic acids, phenolic diterpenes, flavonoids, catechins, procyanidins and anthocyanins) which are important dietary factors (Alam et al. 2011). The pharmacological effect of the *W. somnifera* roots is related to active ingredient, *withanolides*, which has therapeutic applications, antibiotics and antitumor and anxiolytic antidepressant remedy in Indian traditional medicine (Bhattacharya et al. 2000). Patwardhan et al. (1998) claimed that leaves (bitter taste) are recommended in case of fever, painful swelling and ophthalmitis. Moreover, other parts of the plant revealed antiserotogenic, anticancer and anabolic properties and exhibited beneficial effects in treatment of arthritis, stress and geriatric problems (Alam et al. 2011; Mirjalili et al. 2009).

Dong quai root is used as a spice, tonic or medicine in China, Korea and Japan (Mei et al. 1991; Ross 2001), and in dietary supplements or as an ingredient of cosmetics available in China, USA and Europe. Its medicinal value has been demonstrated in numerous clinical trials, preclinical studies and traditional practices (Mei et al. 1991; Ross 2001), especially for stabilizing endocrine women, in menopause or menstruation problems. Analytical studies of Dong quai revealed the presence of over 70 compounds; such as essential oils, polysaccharides, ferulic acid and other phenolic acids, ligustilide, phthalides, vitamins and micro- and macroelements (Ross 2001; Huang et al. 2008; Lao et al. 2004; Lu, et al. 2005).

Ginseng is one of the most popular herbal remedies. The biochemical and pharmacological ginseng activity as: antiaging, antidiabetic, anticarcinogenic, analgesic, antipyretic and antistress, antifatigue, tranquilizing remedies has been reported (Jung et al. 2002). In the case of Korean ginseng (P. ginseng C.A. Meyer), the active components are mainly saponins (ginsenosides). However, non-saponin components have recently received a great attention due to their antioxidant, anticancer, antidiabetic and immunomodulating activity. The non-saponin components in ginseng are flavonoids (Ma et al. 2005), triterpenoids (Agarwal et al. 2005), and phenolic acid (salicylic acid, vanillic acid, and p-coumaric acid) (Jung et al. 2002). The latter one is principal antioxidant components (Jung et al. 2006).

To the best of our knowledge, there is a lack of reports in the literature on the antioxidants, phytochemicals or toxic ingredients in Dong quai, Ashwagandha, whereas for *Panax ginseng* are few. *Panax ginseng*, Ashwagandha and Dong quai are marketed as dietary supplements or their ingredients, but due to EU legislation, producers are not obliged to provide data on the chemical content. The positive impact on human health and bioavailability claimed for flavonoids and phenolic acids in botanical products and natural plant sources, as well as potentially toxic of alkaloids and their negative effects, prompts their qualitative and quantitative analysis.

Therefore, for the effective application of ginsengs and other medicinal herbs in dietary supplements, qualitative and quantitative data on the antioxidants and phytochemicals seems to be essential. Moreover, increasing consumption of dietary supplements and teas containing herbs in Poland and Europe, is determined by their health promoting properties (e.g., the presence of antioxidants), availability and marketing policy. Hence, the main aim of the presented work is analysis of dietary supplements and pure herbs for the content of pharmacologically active and potentially toxic compounds.

Mycotoxins are well-known group of extremely toxic components produced by certain species of fungi, which can be present in the studied medicinal plants and dietary supplements. We focused on the determination of aflatoxins (AF) (produced by *Aspergillus flavus* and *Aspergillus parasiticus*), and ochratoxin A (OTA) (*Penicillium* and some species of *Aspergillus*). These mycotoxins are highly carcinogenic, mutagenic and teratogenic components which pose serious health and economic concerns to humans; hence they should be monitored in food. Pre- and post harvest stages and storage conditions (temperature, relative humidity and duration), product processing technology or agricultural practices have critical roles in the production of mycotoxins. Moreover, many mycotoxins are extremely stable and cannot be degraded by simple psychical procedures (e.g., heat treatment) (Bryden 2012).

Therefore, the main aim of this study was to determine the contamination of mycotoxins, such as aflatoxin and ochratoxin A, as well as hazardous alkaloids. Additionally, cytotoxicity evaluation was performed using MTT test (using (3-(4,5-dimethylthiazol-2-yl)-2,5-diphenyltetrazolium salt). In this work, LC–MS/MS method involving ethanolic extraction was applied for the determination of flavonols: Q—quercetin, Qc—quercitrin, Hy—hyperoside, R—rutin, Rh—rhamnetin, Kl—kaempferol, and My—myricetin, phenolic acids: GA—gallic acid, CA—caffeic acid, ChA—chlorogenic acid, FA—ferulic acid, pCA—*p*-coumaric acid, hBA—*p*-hydroxybenzoic acids, purine alkaloids: Cf—caffeine, Tb—theobromine, Tf—theophylline, indole alkaloids: Ha—harmine, Hae—harmane, Hl—harmol, Y—yohimbine, Bru—brucine, St—strychnine and isoquinoline alkaloids: Em—emetine, Ber—berberine, Ns—noscapine, Pv—papaverine in the *Panax ginseng*, Ashwagandha and Dong quai. Additionally the total content of polyphenols (TPC) was determined by the Folin–Ciocalteu method, whereas antioxidant activity (AA) by FRAP and iron(III)-phenanthroline antioxidant assays.

## Experimental

### Materials and methods

Ashwagandha (Aa), Dong quai (DQ) and *Panax ginseng* (PG) dried, powdered materials were purchased from Standard (Lublin, Poland), while dietary supplements containing ginsengs in the form of pills (denoted as DS-Aa, DS-DQ, DS-DQ-S, DS-PG) were bought in a local pharmacy.

### Extraction procedure

The ground, dried samples (2.00 ± 0.01) g of *Panax ginseng*, Ashwagandha, Dong quai and dietary supplements were extracted three times with 20 mL of ethanol (for phytochemicals and alkaloids studies) and water (for TPC and AA) at 45 °C, in an ultrasonic water-bath shaker for 1 h. Next, extracts were centrifuged (4500 min^−1^, 15 min), supernatant separated and used for analysis.

#### Determination of selected alkaloids, flavonoids and phenolic acids

Chromatographic separations of alkaloids, flavonols and phenolic acids were performed using electrospray ionization (ESI)-liquid chromatograph mass spectrometry (LCMS-8030 SHIMADZU, Japan) with photodiode multi-wavelength Prominence (SPD-M20A, SHIMADZU, Japan) and MS Nexera (SHIMADZU, Japan) detector supported by Shimadzu LabSolutions v. 5.60 system software.

LC–MS/MS analyses were performed using a reverse phase column (Kinetex, 2.6 u C18 100A, 100 × 3.0 mm, Phenomenex) at the following parameters: nebulizing gas: 1.5 L min^−1^; drying gas: 15 L min^−1^; desolvation line temperature: 250 °C; and heat block temperature: 400 °C. Temperature of the column and cell detector was set at 25 °C. Positive ionization MRM procedure was applied for all studied alkaloids, while negative for phenolic acids and flavonols. The mobile phase was composed of methanol (phase A) and 2% acetic acid (phase B), total flow rate of 0.4 mL min^−1^, and the gradient conditions were (0–5 min from 2 to 45% B, 5–8 min from 45 to 55% B, 8–12 min from 55 to 100% B and maintain 100% B for next 2 min) resulting in properly resolved and shaped peaks in separation time below 15 min. The sample injection volume was 1 µl.

Calibration curves for studied phytochemicals were calculated by standards solutions method (concentration range 0.001–0.10 or 0.10–1.00 mg L^−1^) in five repetitions. The LC–MS/MS method was validated for linear dynamic range, detection limit (DL), quantification limit (QL), linearity, intra-day and inter-day precision. The LOD and LOQ were estimated as three and ten times the signal-to-noise ratio (S/N), respectively. The reproducibility was determined by standard solution injection five times in one day experiment, whereas for inter-day variation (repeatability), the standard solution was analyzed twice a day on three consecutive days. Calculated relative standard deviations (RSD%) were in the range 0.54–2.79% for reproducibility and 0.83–3.22% for repeatability. The recoveries obtained for studied phytochemicals ranged from 97.7 to 100.5%, indicating good accuracy of the method.

#### Evaluation of antioxidant activity

FRAP method (Ferric ion reducing antioxidant parameter) was performed according to Filipiak-Szok et al. procedure (Filipiak-Szok et al. 2014). Five calibration curves were prepared using working solutions of Trolox (TE) (2.00–25.00 μM) in ethanol resulting in the linear equation: *y* = (0.0451 ± 0.0003)*x* − (0.0362 ± 0.0041) (*R* ^2^ = 0.9997, RSD_slope_ = 1.29%, DL = 0.50 μM and QL = 1.52 μM). FRAP values were expressed as μmol TE/g of dry mass (d.m.) ± CI (confidence interval), whereas determined mean recovery was (97.81 ± 0.15) %, and the molar absorption coefficient (3.92 ± 0.46) × 10^4^ dm^3^ mol^−1^ cm^−1^.

Antioxidant activity was also determined by iron(III)-phenanthroline ([Fe(phen)_3_]^2+^) method, modified according to Ozyurek et al. (2007). In this procedure, Trolox, Aa, DQ, PG and dietary supplements extracts (0.1–0.5 mL) were mixed with 1 mL 0.025 M FeCl_3_ solution in 0.5 M HCl, 1 mL 0.05 M ethanolic solution of 1,10-phenanthroline. pH was set in the range 5.5–6.0. Solutions were mixed, incubated for 30 min at 70 °C in the dark, resulting red–orange complex. The absorbance was measured at 510 nm against blank. Five calibration curves were prepared using Trolox working solutions (5.00–45.00 μM) in ethanol resulting in the linear equation: *y* = (0.0238 ± 0.0002)*x* − (0.0050 ± 0.0058) (*R* ^2^ = 0.9995, RSD_slope_ = 1.35%, DL = 1.11 μM and QL = 3.37 μM). Mean recovery was (98.03 ± 0.13) %, and molar absorption coefficient (2.34 ± 0.05) × 10^4^ dm^3^ mol^−1^ cm^−1^. Results were expressed as μmol TE/g d.m. (dry mass) ± CI (confidence interval).

Spectrophotometric measurements were performed with a spectrophotometer UV–VIS HELIOS α, (Unicam, England) with 1 cm quartz cell was used.

#### Determination of total phenolic compounds (TPC)

The Folin–Ciocalteu reagent in basic medium (saturated sodium carbonate solution) was added to the standard solution of gallic acid (1.00–10.00 mg L^−1^), mixed, filtered, incubated at room temperature for 1 h and absorbance was measured at *λ* = 725 nm. Calibration curves equation (based on five repetitions): *y* = (0.1307 ± 0.0010)*x* − (0.0337 ± 0.0058), (*R* ^2^ = 0.9995, RSD_slope_ = 1.56%, DL = 0.25 mg L^−1^, QL = 0.75 mg L^−1^). The results were expressed as mg GAE (gallic acid equivalent) per g d.m. ± CI. The accuracy was determined by mean recoveries (97.64 ± 0.235) %, while the molar absorption coefficient was (2.02 ± 0.10) × 10^4^ dm^3^ mol^−1^ cm^−1^ (Filipiak-Szok et al. 2014). The same procedure was used for plant and dietary supplement extracts analysis.

### Mycotoxins determination

#### Aflatoxins

12.5 g of plants and dietary supplements were used for samples preparations according to Błajet-Kosicka et al. (2014) and Grajewski et al. (2012) procedures. The content of AF was determined by HPLC using a post-column derivatization with Kobra^®^.

#### Ochratoxin A

12.5 g of plants and dietary supplements samples were homogenized with 50 mL of ACN:H_2_O (60:40) for 2 min. Next, samples were prepared according to previously reported and changed procedures by Błajet-Kosicka et al. (2014) and Grajewski et al. (2012). In Table 1 analytical parameters for mycotoxins analysis is presented.

**Table 1:** Mycotoxins analysis

	LOD (μg kg^−1^)	LOQ (μg kg^−1^)	Linearity range (μg kg^−1^)	Spiking level (μg kg^−1^)	Recovery ± SD (%) *n* = 3
OTA	0.13	0.40	0.51–151.2	12.6	97.9 ± 0.9
AF G_1_	0.11	0.33	0.41–40.6	1.62	71.0 ± 5.4
AF G_2_	0.08	0.24	0.10–10.0	2.42	75.9 ± 6.0
AF B_1_	0.06	0.18	0.40–40.2	1.61	80.8 ± 1.8
AF B_2_	0.03	0.09	0.10–10.1	2.41	85.2 ± 1.3

### Mycological analysis

A sample portion (5.0 g) of dry, grounded plants and dietary supplements was placed in a sterile bag within a Stomacher-type homogenizer (BagMixer 400, Interscience, France); 45 mL of sterile dilution fluid were added and the mixture was homogenized for 90 s. The number of fungi (cfu g^−1^) was determined according to PN-ISO-7954 (1999). Samples preparation and analysis were performed by improved procedures described by Mayer et al. (2016) and Błajet-Kosicka et al. (2014).

### Cytotoxicity evaluation

Cytotoxicity evaluation was performed using MTT (3-(4,5-dimethylthiazol-2-yl)-2,5-diphenyltetrazolium salt) test with swine kidney (SK) cells. Swine kidney cells were grown on medium containing the solution of antibiotics (penicillin and streptomycin, Sigma Aldrich) and fetal calf serum (Sigma Aldrich) in a CO_2_ Hera Cell incubator (Heraeus, Germany) (5% CO_2_, 37 °C, humidity at 98%). The samples were prepared according to the improved analytical procedure previously described by Mayer et al. (2016). On the basis of the subsequent steps of dilution cytotoxicity—IC_50_ was determined, which is the sample concentration at which cell proliferation was inhibited by 50% compared to control cells.

### Statistical analysis

One-way ANOVA, followed by Duncan test, were preformed to analyze the significant differences between data at the *p* < 0.05 level using Statistica (Windows software package, version 10.0 PL). ANOVA test and cluster analysis were to create the answer, if analyzed dietary supplements and different ginseng samples derived from Traditional Chinese Medicine and Ayurveda (Ashwagandha, Dong quai, Panax ginseng) can be grouped into different classes.

## Results and discussion

### Phytochemicals content

Results of determination of flavonols, phenolic acids and purine, indole and isoquinoline alkaloids content in Ashwagandha, Dong quai, *Panax ginseng* and dietary supplements are listed in Tables 2 and 3. The analytes on the chromatograms were identified by the retention times and *m*/*z* ratio using the reference standards under the same chromatographic conditions or by spiking the extracts with the reference standards. Due to the molecular complexity of the natural samples extracts, the lack of standards and the detector applied, only selected peaks were identified.

**Table 2:** Flavonols and phenolic acids content in Ashwagandha, Dong quai and Panax ginseng plants and dietary supplements determined by LC–MS/MS

	Content of flavonols [mg 100 g^−1^ d.m. ± CI] (*n* = 3)							Content of phenolic acids [mg 100 g^−1^ d.m. ± CI] (*n* = 3)					
	R	Hy	Qc	My	Q	Kl	Rm	GA	CA	ChA	FA	pCA	hBA
Aa	4.21 ± 0.05^b^	0.82 ± 0.04^a^	5.22 ± 0.13^a^	0.22 ± 0.03^b^	7.21 ± 0.52^a^	0.78 ± 0.08^a^	1.15 ± 0.09^a^	4.02 ± 0.13^a^	1.99 ± 0.12^a^	1.03 ± 0.08^a^	0.55 ± 0.08^a^	0.67 ± 0.09^a^	0.21 ± 0.03^a^
DS-Aa	4.06 ± 0.13^a^	0.88 ± 0.05^b^	5.58 ± 0.21^b^	0.18 ± 0.03^a^	7.84 ± 0.44^b^	0.95 ± 0.11^b^	1.26 ± 0.11^b^	4.55 ± 0.24^b^	2.02 ± 0.14^a^	1.22 ± 0.15^b^	0.61 ± 0.04^b^	0.75 ± 0.09^b^	0.25 ± 0.02^b^
DQ	7.28 ± 0.12^c^	1.51 ± 0.10^c^	2.44 ± 0.07^c^	ND	9.51 ± 0.21^c^	0.11 ± 0.03^c^	2.92 ± 0.09^b^	3.48 ± 0.07^c^	1.66 ± 0.04^a^	0.28 ± 0.03^b^	2.88 ± 0.11^c^	1.89 ± 0.08^c^	0.61 ± 0.04^c^
DS-DQ	6.10 ± 0.10^a^	0.71 ± 0.03^a^	2.08 ± 0.05^a^	ND	7.77 ± 0.22^a^	0.05 ± 0.01^a^	2.76 ± 0.07^a^	3.04 ± 0.05^a^	1.74 ± 0.03^b^	1.06 ± 0.05^c^	2.51 ± 0.08^b^	1.49 ± 0.10^b^	0.47 ± 0.06^b^
DS-DQ-S	6.47 ± 0.12^b^	0.79 ± 0.02^b^	2.21 ± 0.04^b^	ND	8.32 ± 0.49^b^	0.07 ± 0.01^b^	2.71 ± 0.10^a^	3.22 ± 0.13^b^	1.77 ± 0.04^b^	0.22 ± 0.03^a^	2.16 ± 0.09^a^	0.74 ± 0.07^a^	0.26 ± 0.02^a^
PG	2.20 ± 0.11^a^	0.31 ± 0.02^a^	13.07 ± 0.16^a^	ND	1.94 ± 0.01^a^	0.11 ± 0.01^a^	0.19 ± 0.01^b^	2.22 ± 0.14^a^	1.03 ± 0.06^a^	0.58 ± 0.04^a^	3.27 ± 0.14^a^	1.05 ± 0.08^a^	ND
DS-PG	2.32 ± 0.13^b^	0.46 ± 0.03^b^	14.11 ± 0.25^b^	ND	2.08 ± 0.02^b^	0.23 ± 0.03^b^	0.13 ± 0.01^a^	2.40 ± 0.09^b^	1.11 ± 0.05^b^	0.65 ± 0.06^b^	3.25 ± 0.13^a^	1.11 ± 0.12^a^	ND

*R* rutin, *Hy* hyperoside, *Qc* quercitrin, *My* myricetin, *Q* quercetin, *Kl* kaempferol, *Rh* rhamnetin, *GA* gallic acid, *CA* caffeic acid, *ChA* chlorogenic acid, *FA* ferulic acid, *pCA p*-coumaric acid, *hBA p*-hydroxybenzoic acids, *ND* not detected

Different letters (a, b and c) within the same column and pair: plant—dietary supplement indicate significant differences (one-way ANOVA and Duncan test, *p* < 0.05)

**Table 3:** Alkaloids content in Ashwagandha, Dong quai and Panax ginseng plants and dietary supplements determined by LC–MS/MS

	Purine alkaloids			Indole alkaloids						Isoquinoline alkaloids			
	Cf	Tb	Tf	Ha	Hae	Hl	Y	Bru	St	Em	Ber	Ns	Pv
Alkaloids content [mg 100 g^−1^ d.m. ± CI] (*n* = 3)													
Aa	1.22 ± 0.09^a^	0.26 ± 0.03^a^	0.11 ± 0.02^a^	0.26 ± 0.02^a^	0.08 ± 0.01^a^	ND	0.04 ± 0.00^a^	ND	ND	ND	0.41 ± 0.04^a^	0.32 ± 0.05^a^	0.16 ± 0.02^a^
DS-Aa	1.35 ± 0.12^a^	0.31 ± 0.04^b^	0.15 ± 0.02^b^	0.33 ± 0.04^b^	0.11 ± 0.02^b^	ND	0.07 ± 0.01^b^	ND	0.01 ± 0.00	ND	0.57 ± 0.08^b^	0.51 ± 0.06^b^	0.19 ± 0.02^b^
DQ	0.94 ± 0.05^b^	ND	0.08 ± 0.01^a^	0.18 ± 0.02^b^	0.06 ± 0.01^b^	0.06 ± 0.01^a^	ND	0.03 ± 0.00^b^	ND	0.04 ± 0.01^c^	0.12 ± 0.02^c^	ND	0.18 ± 0.02^a^
DS-DQ	0.85 ± 0.09^a^	ND	0.11 ± 0.02^b^	0.13 ± 0.03^a^	0.03 ± 0.00^a^	0.08 ± 0.01^b^	ND	0.02 ± 0.00^a^	ND	0.03 ± 0.00^b^	0.10 ± 0.02^b^	ND	0.18 ± 0.02^a^
DS-DQ-S	0.93 ± 0.04^b^	0.17 ± 0.03	0.13 ± 0.02^c^	0.11 ± 0.02^a^	0.03 ± 0.00^a^	ND	ND	ND	ND	0.02 ± 0.00^a^	0.07 ± 0.01^a^	ND	0.19 ± 0.03^a^
PG	1.42 ± 0.11^a^	0.32 ± 0.03^a^	ND	0.29 ± 0.03^a^	ND	ND	0.07 ± 0.01^a^	0.05 ± 0.01^a^	ND	ND	0.25 ± 0.02^a^	ND	ND
DS-PG	1.55 ± 0.13^a^	0.43 ± 0.03^b^	ND	0.32 ± 0.04^b^	ND	ND	0.09 ± 0.01^b^	0.07 ± 0.01^b^	ND	ND	0.28 ± 0.03^b^	ND	ND

*Cf* caffeine, *Tb* theobromine, *Tf* theophylline, *Ha* harmine, *Hae* harmane, *Hl* harmol, *Y* yohimbine, *Bru* brucine, *St* strychnine, *Em* emetine, *Ber* berberine, *Ns* noscapine, *Pv* papaverine, *ND* not detected

Different letters (a, b and c) within the same column and pair: plant and dietary supplement indicate significant differences (one-way ANOVA and Duncan test, *p* < 0.05)

The contents of phenolic acids, flavonols and alkaloids, thereby obtained were statistically analyzed by one-way ANOVA followed by the Duncan test (Tables 2, 3). The results were compared against each other in column in pair: plant and dietary supplement for individual phytochemicals (e.g., Aa and DS-Aa for R) and significant differences were allocated a different superscript (*a*–*c*) and similarities by the same letter in superscript. It is noteworthy that, in many cases, the phytochemicals content in the samples in the study exhibited significant differences. However, for some phenolic acids, flavonols and alkaloids the results obtained for plant extracts did not differ from the supplement extracts (e.g., DS-DQ and DS-DQ-S for Rm, CA, Ha, and Hae, Aa and DS-Aa for CA, Cf as well as PG and DS-PG for FA, pCA and Cf). For Dong quai, we observed similarities in two dietary supplements DS-DQ and DS-DQ-S, and what is important, for papaverine we observed similarities in three samples of DQ.

Among flavonols, quercetin (1.94–9.51 mg 100 g^−1^) and rutin (2.20–7.28 mg 100 g^−1^) were predominant, whereas in PG and DS-PG quercitrin was determined in a high concentration level. However, Kl and Hy were indicated in the smallest concentration. Myricetin was detected only in Ashwagandha and dietary supplements containing Aa. Analysis of phenolic acids in studied samples indicates gallic acid (4.02 mg 100 g^−1^ for Aa) as predominant constituents, while in PG the highest concentration was obtained for ferulic acid (3.27 mg 100 g^−1^). However, in the PG and DS-PG samples p-hydroxybenzoic acid was not detected In the Aa, DQ and PG extracts, other phytocompounds were determined in minor amounts. In many cases, we observed, lower concentrations of flavonols, phenolic acids and alkaloids in the Aa, DQ and PG extracts were noted in relation to dietary supplements. The same situation we obtained for examined alkaloids. Purine alkaloids occur most often, moreover, isoquinoline alkaloids are less common than indole one. In DQ and DS-DQ was not detected, whereas PG and DS-PG do not contain theophylline. The predominant alkaloids is harman (from indole group) and berberine (from isoquinoline one). In the studied plants and dietary supplements, alkaloids were revealed at the lower concentration in comparison to phenolic acids or flavonols. On the other hand, toxic strychnine was determined in DS-Aa and this result reveals the danger of consuming dietary supplements and the need to analyze these products.

**Fig. 1:**
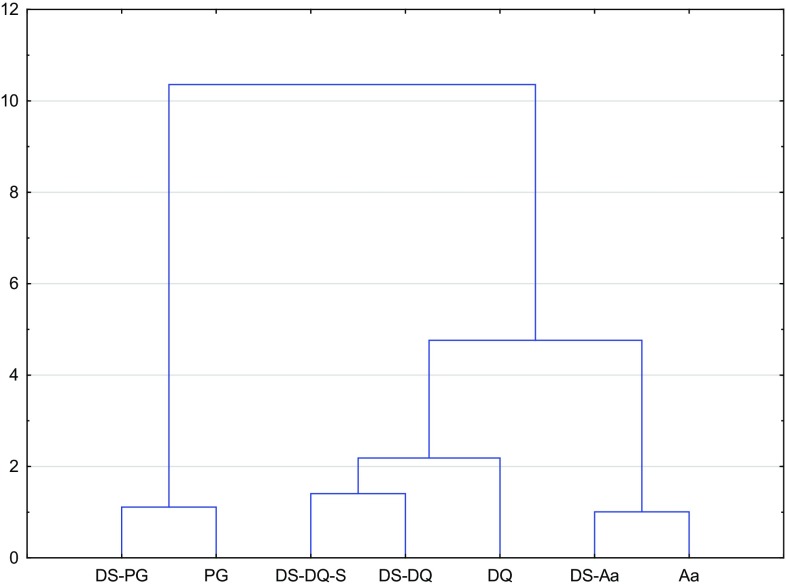
Cluster analysis of Aa, DQ, PG and dietary supplements

Cluster analysis results are presented at Fig. 1. Plants and dietary supplements containing this plant revealed some similar properties (Aa and DS-Aa, DQ and DS-DQ, DS-DQ-S and PG and DS-PG). First of all, Aa and DS-Aa were the closest, whereas DQ and dietary supplements of DQ were the most different. Moreover, the branch of DQ and Aa is close, but PG formed one separate branch. It is noteworthy that this variability can be explained by different type of studied ginseng: Indian ginseng, female ginseng and *Panax ginseng*. Moreover, the influence of genetics, agronomic, and environmental factors, different geographical region, different growing conditions, storage and purchasing sources, may affect the type and concentration of antioxidants and phytochemicals. On the other hand, extraction procedure and analyzing method can cause the differences.

To the best of our knowledge, the literature data on the content of flavonols and phenolic acids in Dong quai, Ashwagandha and Panax ginseng are limited. However, alkaloids are difficult to compare, because of lack of information about it. Huang et al. (2008) determined ferulic, *p*-coumaric, vanillic, caffeic, *trans*-cinnamic, nicotinic, protocatechuic and phthalic acids by HPLC in DQ. Authors determined ferulic acid (15.1–18.9 mg mL^−1^, as a major constituents, whereas concentration of other phenolic acids were below 2.46 mg mL^−1^). Lu et al. (2005) analyzed free ferulic acid and determined total ferulic acids in DQ (0.21–1.43 mg g^−1^). Phenolic acids, flavonoids were determined in methanolic extracts of *W. somnifera* fruits, roots and leaves by Alam et al. (2011) were in the range of 17.80 ± 5.80 and 32.58 ± 3.16 mg/g (dry weight, respectively). Moreover, eight polyphenols (Gallic, syringic, benzoic, p-coumaric and vanillic acids as well as catechin, kaempferol and naringenin) have been identified by HPLC in Ashwagandha. Among all the polyphenols, catechin was detected in the highest concentration (13.01 ± 8.93 to 30.61 ± 11.41 mg/g). 12 different phenolic acids as free, esterified, and insoluble-bound forms were identified in Korean ginsengs by Jung et al. (2002). Total phenolic acids in white and red ginsengs were 27.2 and 26.8 mg 100 g^−1^, respectively. Ferulic acid (*cis* and *trans* isomers) was the major of free phenolic acids and also in insoluble-bound phenolic acids (Jung et al. 2002). Total phenolics, flavonoids and ascorbic acid contents in various solvent extracts of ginseng leaves were also been described by Jung et al. (2006). Authors reported that ethanol extracts from ginseng leaves contained more phenolics (2333.2 mg/100 g) and flavonoids (1199.1 mg/100 g) than other solvents extracts. According Jung et al. (2006), flavonol aglycones of quercetin and kaempferol were quantified by HPLC method in concentration ranges 132.3–175.6 and 333.0–514.4 mg/100 g, respectively.

### Antioxidant activity and total phenolic content

Results of antioxidant activity (AA) and total phenolic content (TPC) determination are listed in Table 4. The highest value of TPC was observed for PG (8.67 ± 0.18 mg GAE g^−1^ d.m.), but the lowest for DS-DQ (2.75 ± 0.0.09 mg GAE g^−1^ d.m.). Moreover, AA results ranging from 18.87 to 54.16 µmol TE g^−1^ (DS-DQ and PG, respectively) by FRAP method, and 19.82–54.79 µmol TE g^−1^ (DS-DQ and Aa, respectively) by [Fe(phen)_3_]^2+^ procedure. The results indicated that in many cases, the [Fe(phen)_3_]^2+^ value were higher than FRAP. It is also worth noting that the dietary supplements with Aa, DQ and PG exhibited smaller values of AA and TPC than the plant extracts.

**Table 4:** Antioxidant activity and TPC in Aa, DQ i PG and dietary supplements

	Total (TPC) [mg GAE g^−1^ ± CI d.m.] (*n* = 5)	Antioxidant activity (AA) [µmol TE g^−1^ ± CI d.m.] (*n* = 5)	
		FRAP	[Fe(phen)_3_]^2+^
Aa	5.22 ± 0.13^b^	53.14 ± 0.88^b,x^	54.79 ± 1.25^a,y^
DS-Aa	4.94 ± 0.16^a^	52.16 ± 1.11^a,x^	54.56 ± 1.13^a,y^
DQ	3.98 ± 0.60^c^	46.50 ± 1.29^c,x^	48.02 ± 1.76^b,x^
DS-DQ	2.75 ± 0.09^a^	18.87 ± 0.77^a,x^	19.82 ± 1.94^a,y^
DS-DQ-S	3.00 ± 0.12^b^	19.62 ± 0.79^b,x^	20.59 ± 4.15^a,y^
PG	8.67 ± 0.18^b^	54.16 ± 1.24^a,x^	53.14 ± 1.73^a,x^
DS-PG	8.62 ± 0.13^a^	54.09 ± 1.79^a,y^	52.66 ± 2.17^a,x^

Different letters (a, b and c) within the same column and pair: plant and dietary supplement, whereas x and y with the same line, indicate significant differences, (one-way ANOVA and Duncan test, *p* < 0.05)

The superscripts in Table 4 are in accordance with those received by one-way ANOVA followed by the Duncan test and represent the differences between the results obtained. The results are compared in columns-pair: plant and dietary supplement (superscripts *a*-*c*) and in rows, results of AA by FRAP and [Fe(phen)_3_]^2+^ procedure (superscripts *x, y*). Similarities were marked by the same letter in superscript. For TPC we observed significant differences, but for AA by [Fe(phen)_3_]^2+^ method we detected similarities in every one of samples. In addition, the similarities between FRAP and [Fe(phen)_3_]^2+^ results we observed only for DQ and PG extracts, other values vary within the same samples.

Regression analysis was performed for correlations between TPC and AA by two method based on reduction of Fe(III) to Fe(II) of Aa, DQ and PG extracts, as well as dietary supplements. Good correlation for TPC and FRAP, and TPC and [Fe(phen)_3_]^2+^ (*r* = 0.76 and *r* = 0.70, respectively, *p* < 0.05 level) was observed.

Alam et al. (2011) described that all of the three different plant parts of Aa presented strong DPPH radical scavenging activities (59.16 ± 1.20 to 91.84 ± 0.38%). The DPPH and lipid peroxidation inhibitory activities of DQ extracts were also reported by Huang et al. (2008) (76.8 ± 2.3) % for ethanolic extract of DQ). Kang et al. (2007) reported the total phenolic contents of American ginseng (AG) and Korean ginseng (KG) were 23.1 and 19.9 mg g ^−1^ GAE, and these values were increased to 57.0 and 51.3 GAE in heated AG and heated KG, respectively. The increase in total phenolic contents by heat processing is thought to be mediated by the increase of free and conjugated phenolic acid contents due to the release of bound phenolic acids linked with glucosides or amine functionalities by heat treatment. In the DPPH-scavenging activity, tests of ginsengs (Kang et al. 2007) AG and KG scavenged 32 and 9% of DPPH radicals, respectively, and heated-AG and KG more strongly scavenged 81 and 67% of DPPH radicals, respectively, at the concentration of 100 mg/mL.

### Mycotoxins and mycological analysis

Results of mycological analysis and mycotoxins are listed in Tables 5 and 6. In the studied plants and dietary supplements, total number of fungi (the sum of mold and yeast) was lower than 69 CFU g^−1^. The most popular mold in examined samples was *Cladosporium* (detected in Aa, DS-Aa, DQ and DS-PG). *Aspergillus* was identified in supplements: DS-DQ and DS-PG, in addition *Penicillium* was distinguished in Aa, *Eurotium* in DS-DQ and 100% *Rhizopus* in PG. Some molds can produce mycotoxins; hence identification of mold species is very important.

**Table 5:** Results of mycological analysis

Samples	Total number of fungi [CFU g^−1^]	Total number of mold [CFU g^−1^]	Total number of yeast [CFU g^−1^]	The percentage of identified mold [%]
Aa	<50 C (36, C)	<30 C (21, C)	<20 C (15, C)	71%* Cladosporium* 29%* Penicillium*
DS-Aa	<10 C (3, C)	ND in 0.1 g	<10 C (3, C)	100%* Cladosporium*
DQ	<10 C (3, C)	<10 C (3, C)	N.D. in 0.1 g	100%* Cladosporium*
DS-DQ	<100 C (69, C)	<10 C (6, C)	<100 C (63, C)	50%* Eurotium* 50%* Aspergillus*
PG	<50 C (23, C)	<50 C (20, C)	<10 C (3, C)	100%* Rhizopus*
DS-PG	<100 C (65, C)	<20 C (15, C)	<50 C (48, C)	60%* Aspergillus* 40%* Cladosporium*

*CFU* colony-forming unit, *C* colony, *ND* not detected

**Table 6:** Results of mycotoxins determination

Samples	Mycotoxins	
	Ochratoxin A (μg kg^−1^)	Aflatoxins (μg kg^−1^)
Aa	3.06	ND
DS-Aa	2.47	**B**_**2**_ **—0.14** B_1_, G_1_, G_2_—ND
DQ	5.83	ND
DS-DQ	1.72	ND
PG	3.20	ND
DS-PG	3.20	ND

*ND* not detected

Our studies revealed the presence of ochratoxins A (OTA) in every studied samples (from 1.72 to 5.83 for DS-DQ and DQ, respectively) and aflatoxins only in DS-Aa (B_2_—0.14 μg kg^−1^]. There are several regulations for total aflatoxin or ochratoxin A limitation in food (Commission Regulation (EU) No. 1881/2006, and No. 165/2010), especially in cereal grains, spices, dried fruits, nuts or dietary foods for special medical purposes. Total number of aflatoxins in grain or dried fruits intended for human consumption should be less than 4 μg kg^−1^, but for nuts and some spices (e.g., curcuma, ginger) less than 10, on the other hand, for OTA in spices less than 15 μg kg^−1^. Unfortunately, for dietary supplements, producer is obligated to present only mycological analysis, but law does not require the analysis of mycotoxins contamination. Mycotoxins consist of a large group of extremely toxic components which are produced by certain species of fungi. It is worth noting that the problem of mycotoxins contamination of food is wide and very important, hence European Food Safety Authority (EFSA) has recently supported research to evaluate the potential risk resulting from food because of mycotoxins. Contamination of food and feed mycotoxins is one of the main concerns of food safety researchers, but these analyses are not carried out for dietary supplements and plant material, hence the difficulty in comparison to results obtained by other authors.

Trucksess et al. (2009) in ginger supplements by RP-HPLC method determined the total content AF—7.34 and 1.93 ng g^−1^ OTA, while the Prado et al. (2012) in the different herbs used in medicine (e.g. *Valeriana officinalis*) detected AF at the level of 2.04–3.38 ng g^−1^. In addition, AF was marked in the variety of food supplements with grape at level 1.52–32.00 ng kg^−1^ (Solfrizzo et al. 2015).

Impact on the development of mycotoxins are: improper storage conditions (humidity, inadequate temperature), but also processing technologies, and processing of raw materials. Because of toxic properties of mycotoxins, they should be analyzed in medicinal, herbal products or dietary supplements intended for human consumption.

### Cytotoxicity evaluation

**Fig. 2:**
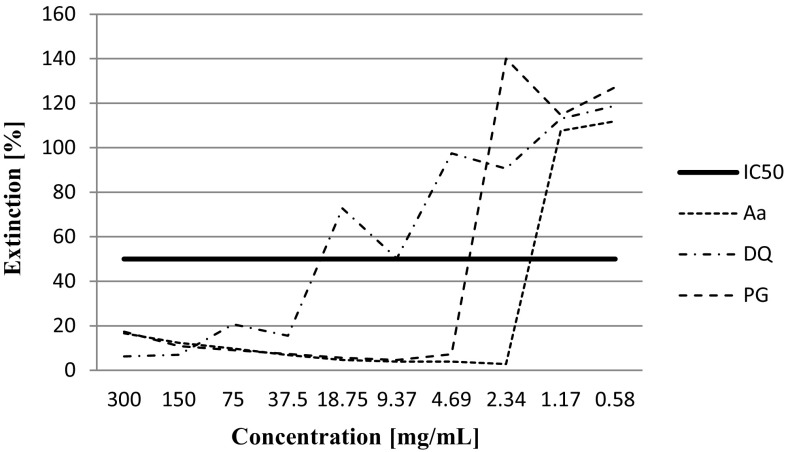
Results of MTT cytotoxicity test on plant extract (Aa, DQ and PG)

Result of cytotoxicity analysis is listed in Table 7 and Fig. 2. MTT cytotoxicity test revealed that extracts of studied plants and dietary supplements exhibited medium or high cytotoxicity (IC_50_ from 9.37 to 1.17 mg mL^−1^ for DS-DQ and DS-Aa, respectively). Low level of cytotoxicity was observed only for Dong quai. For Aa and DS-Aa, we observed the highest level of cytotoxicity among analyzed samples. Overall, this study evaluate that methanolic extracts of Ashwagandha, *Panax ginseng* and dietary supplements containing these plants have potential cytotoxic activity, indicating the presence of cytotoxic compounds in these extracts. This study provides only basic data, but our previous studies focused on phytochemicals, biologically active substances from these extracts (especially alkaloids) can correlate with cytotoxic activity.

**Table 7:** Results of cytotoxicity test

Samples	Step	IC_50_ (mg mL^−1^)	Level of cytotoxicity
Aa	8	2.34	High
DS-Aa	9	1.17	High
DQ	4	37.5	Low
DS-DQ	6	9.37	Medium
PG	7	4.69	Medium
DS-PG	7	4.69	Medium

## Conclusion

This study focuses not only on methods for the evaluation of antioxidants (phenolic acids and flavonols), toxic ingredients (alkaloids, mycotoxins), cytotoxicity and antioxidant activity, but also on a comparison of studied parameters levels in different sources of ginseng: Ashwagandha, Dong quai and *Panax ginseng*. Simultaneous determination of seven flavonols, six phenolic acids and 13 alkaloids with good accuracy (recovery 97.7–100.5%) and precision (RSD < 3.22%) was possible using LC–MS/MS technique. The examined plant extracts are rich in antioxidants, whereas concentration of toxins and alkaloids is rather low, hence these plants can be a potential source of natural phytochemicals and nutrients.

*Withania somnifera*, *Panax ginseng or Angelica sinensis* are used widely in Ayurvedic or alternative medicine because of their therapeutic values, connected especially with bioactive components. However, our study revealed that these plants or dietary supplements may contain toxic composition (e.g., alkaloids and mycotoxins). In addition, the presence of mycotoxins (especially aflatoxin and ochratoxins A) and yeast as well as different species of mold or strychnine in a supplement DS-Aa, suggest that studied medicinal plants and dietary supplements can exert a negative impact on health. MTT cytotoxicity test exhibited that Aa, DS-Aa, PG, DS-PG and DS-DQ have potential cytotoxic activity. Because of the fact that herbal medicines make up an important component of the trend toward not only alternative medicine, information on the various properties, chemical composition, potentially toxic components presented here can be useful in the production of dietary supplements or in quality control.
